# ‘Like a rug had been pulled from under you’: The impact of COVID‐19 on teachers in England during the first six weeks of the UK lockdown

**DOI:** 10.1111/bjep.12381

**Published:** 2020-09-25

**Authors:** Lisa E. Kim, Kathryn Asbury

**Affiliations:** ^1^ Department of Education University of York UK

**Keywords:** coronavirus, COVID‐19, lockdown, qualitative, school closure, teacher stress, teachers, thematic analysis

## Abstract

**Background:**

On 20 March 2020, in response to COVID‐19, UK schools were closed to most pupils. Teachers were required to put remote teaching and learning in place with only two days’ notice from the government.

**Aims:**

The current study explores teachers’ experiences of this abrupt change to their working practices, and during the 5–6 weeks that followed.

**Sample:**

Twenty‐four teachers from English state schools were interviewed, representing mainstream primary and secondary schools and a range of years of experience and seniority.

**Methods:**

Participants were asked to tell stories of three key scenes during the first 5–6 weeks of lockdown: a low point, a high point, and a turning point. A reflexive thematic analysis of their narratives was conducted.

**Results and Conclusions:**

Six themes were identified: *uncertainty*, *finding a way*, *worry for the vulnerable*, *importance of relationships*, *teacher identity,* and *reflections*. Teachers’ narratives suggest that, after an initial period of uncertainty they settled into the situation and found a way forward, supported by strong relationships. However, they remain extremely worried about the most vulnerable pupils and want more joined‐up thinking from the government on how to support them effectively, along with clarity from policymakers to enable planning ahead. Teachers reflected on how to use their learning during this period to improve pupils’ experiences of education post‐COVID‐19, and on how aspects of shared teacher identity have worked as stressors and coping mechanisms. These initial interviews form the baseline for a longitudinal interview study of teachers’ experiences of COVID‐19 in England.

## Background

On 20 March 2020, after the announcement of a national lockdown, England’s schools were closed to all pupils other than key workers’ children and those deemed vulnerable. By this date, schools in 137 countries were closed as a result of COVID‐19 (The World Bank, 2020). These decisions have affected 1.5 billion students around the world and 11.2 million primary and secondary school pupils in the UK (The World Bank, 2020; UNESCO, 2020a).

School closures are not new in history. The influenza pandemic in 2009 saw some schools closing around the world, including in England (Jackson *et al*., 2014). Reports on the impact of school closures as a result of a pandemic have focused on whether such closures are effective in controlling the spread of disease (Rauscher, 2020; Viner *et al*., 2020), and their effects on pupils’ learning and well‐being (Asbury *et al.*, 2020; Colao et al., 2020). Little attention has been paid to the impact school closures have on teachers. This is an important gap given that teachers constitute an essential workforce in all societies (Schleicher, 2018).

### Teacher stress and coping

Confusion and stress for teachers has been identified by UNESCO (2020b) as one of 13 adverse consequences of school closures and is linked to the abruptness of the closures, uncertainty about how long they will last, and low familiarity with remote education. Teacher stress, the experience of unpleasant job‐related emotions associated with the depletion of psychological resources (Kyriacou, 2001; Lazarus & Folkman, 1984), has long been a prevalent topic of discussion among educational practitioners, policymakers, and researchers (OECD, 2020b). This attention may be due to the understanding that prolonged experiences of stress can lead to teachers experiencing burnout which, in turn, is associated with both lower confidence in their ability to do their job (Burić & Kim, 2020; Skaalvik & Skaalvik, 2010) and intention to quit their job (Burić & Kim, 2020; Weisberg & Sagie, 1999). Before the current pandemic, the most prominent job stressors for teachers were workload and behaviour management (Catalán *et al*, 2019; Boyle *et al*., 1995; Kokkinos, 2007). Recognizing these difficulties, the UK Department for Education (DfE) has supported initiatives to address the challenges of teacher workload (Churches, 2020; Walker *et al*., 2019) and has outlined a plan to improve the situation (Department for Education, 2019a). Given the major changes in the nature of day‐to‐day teaching during COVID‐19 (Reimers & Schleicher, 2020), the current study sheds light on whether workload and behaviour management have been replaced by other stressors, and how these new stressors may have affected teachers.

COVID‐19 has necessitated many changes in society, including those in education that are likely to be cognitively and emotionally taxing for teachers. Engaging in remote teaching has clearly been one of the most prominent changes required of teachers (Department for Education, 2020a). This challenge has been exacerbated by pupils’ varying levels of access to online technology (OECD, 2020a) and willingness to engage (Borup *et al*., 2020). Furthermore, some teachers may have competing responsibilities, such as home schooling their own children, caring for vulnerable family members, and/or managing their own mental health. All of this runs parallel to teaching their pupils remotely and continuing the non‐teaching elements of their job, and the combination of these circumstances can present a potentially highly stressful situation for teachers. Although all teachers may have experienced similar stressors, stress and coping theories (Lazarus & Folkman, 1984; Parker & Endler, 1996) recognize that individuals can appraise and interpret the same stressors in different ways. Thus, we examine how teachers identify and describe the potential stressors they experienced during the initial phase of lockdown.

When experiencing stress, individuals use coping strategies to alleviate or ameliorate the source or the experience of stress. According to one of the dominant inventories of coping, the COPE scale (Carver *et al*., 1989), coping strategies can largely be organized into two groups: problem‐focused strategies (intended to alter the source of the stress; e.g. planning for the future) and emotion‐focused strategies (intended to alter the emotional experience of stress; e.g. seeking emotional support). For example, workload can often instigate teachers’ use of emotion‐focused coping strategies while student misbehaviour is more likely to lead to problem‐focused coping strategies (Pogere *et al*., 2019). During COVID‐19, with its unique practical and emotional challenges, it is likely that these stressors will trigger the use of both groups of coping strategies. However, we anticipate that emotion‐focused coping strategies may be particularly prominent as many of the stressors are beyond individual teachers’ control, thus requiring management of their affective responses. As such, the current study explores whether teachers have adapted to the professional changes necessitated by COVID‐19 in ways that would be predicted by existing empirical and theoretical research.

### Core values in teaching

Removing teachers from their usual work environments and asking them to work in new ways inevitably raises the question of what it means to be a teacher during this time. Teacher identity is recognized as a multifaceted construct but there is no clear consensus on its definition or its theoretical basis (Beauchamp & Thomas, 2009). From an identity theory perspective (Brenner *et al*., 2018; Burke & Stets, 2009), for example, teacher identity is something that one develops and then preserves as part of role identity. A review of teacher identity measures outlined that these measures assess six components (Hanna *et al*., 2019); namely, self‐image (view of self as a teacher), motivation (reason for teaching), commitment (dedication to the profession), self‐efficacy (ability to carry out activities as a teacher), task perception (understanding the task of a teacher), and job satisfaction (satisfaction level with the teaching job). Reflections on critical incidents, such as a global pandemic, are helpful in eliciting important components of a profession (Flanagan, 1954), making COVID‐19 a unique opportunity to gain new insights into teacher identity. As such, we aim to contribute to the ongoing discussions about what it means to be a teacher by examining teachers’ descriptions of their professional experiences during the initial phase of lockdown in England in Spring 2020.

Tools designed to capture qualities of effective teachers often include teachers’ ability to connect with pupils, parents, and colleagues (see Goe *et al*., 2008 for a review). The teaching profession is inherently a caring one (Lavy & Naama‐Ghanayim, 2020) and positive teacher–student relationships are an important source of job satisfaction, providing meaning and purpose and an underpinning fidelity to the profession (Spilt *et al*., 2011; Veldman *et al*., 2013). Moreover, relationships with colleagues and pupils’ parents are known to be important elements of teaching and teacher identity (Hargreaves, 2001; Hughes & Kwok, 2007). Therefore, a shift to remote education, which disrupts or changes the nature of interpersonal connections, might be expected to affect teachers’ sense of professional identity.

In addition to prioritizing and appreciating relationships, effective teachers are able to reflect on their own practice (Hopkins & Stern, 1996). In fact, this capacity to reflect is actively developed during teacher education (Francis, 1997; Körkkö *et al*., 2016) and throughout teachers’ careers (Hen & Sharabi‐Nov, 2014; Tsui, 2009). The focus of reflection can vary, ranging from individual teachers’ pedagogical practice through to the wider educational system in which they operate. Given the many changes that teachers have been going through since March 2020, examining the nature of their reflections could provide useful insights into their experiences and their interpretations of those experiences, including how they engage with pupils, parents, and colleagues, as well as their pedagogical approach. Moreover, examining these reflections through self‐stories (McAdams & McLean, 2013) will allow us to take a narrative approach, which can enhance our understanding of their experiences and perceptions.

### Narrative identity theory

Our exploratory study of teachers’ experiences during COVID‐19 is rooted in the theoretical framework of narrative identity (McAdams, 2001; McAdams & McLean, 2013). Narrative identity theory argues that individuals develop and internalize an evolving life story that is rooted in episodic autobiographical memory and provides life with unity and a sense of purpose (McAdams, 2001). Asking participants to share scenes from their life stories enables researchers to ask questions, which are highly relevant during a global pandemic, such as: *how do human beings make narrative sense of adversity?* Empirical research has found that aspects of narrative identity, such as drawing redemptive meanings from difficult experiences and emphasizing agency and exploration in self‐stories, are associated with well‐being and psychological adaptation (Bauer *et al*., 2005). By asking participants to tell stories about their professional experiences during COVID‐19 and to draw meaning from those experiences, we hope to gain valuable insights into how the pandemic is affecting teachers and how it may continue to affect them in the future.

### The current study

The main aim of the current study was to document teachers’ initial experiences of partial school closures and lockdown in England during the COVID‐19 pandemic, in order to gain insight into the changes that occurred in schools and their implications for teachers. Using a narrative identity framework, our study therefore aimed to explore the following question: *What were teachers’ experiences like during the first 5–6 weeks of partial school closures in England due to COVID‐19?*

## Method

### Participants

Twenty‐four teachers in England (11 primary and 13 secondary; 6 male and 18 female) volunteered to take part in a longitudinal interview study of the impact of COVID‐19 on teachers. We specified that all participants should work in mainstream state schools as independent and alternative provision schools face different challenges.

The characteristics of each of the 24 participants can be found in Table 1. Specifically, the participants’ gender (Female/Male) and their school stage (Primary/Secondary) are reported. Additionally, we identify teachers as members of Senior Leadership Teams (SLTs) or as classroom teachers, divided on the basis of years of experience in a similar way to Gu and Day’s (2007) categories: Early Career Teacher (ECT; ≤5 years of experience), Mid‐Career Teacher (MCT; 6–18 years of experience), and Late Career Teacher (LCT; ≥19 years of experience).

**Table 1:** Teachers’ characteristics and their low, high, and turning point scenes

Participant no.	Characteristics	Low	Theme	High	Theme	Turning point	Theme
1	Female Primary SLT	Pupil’s mother calling to say she can’t cope	Worry for the vulnerable	Being in school with key workers'/vulnerable children	Importance of relationships	Decisions about free school meals	Finding a way
2	Female Primary SLT	Notification of domestic violence	Worry for the vulnerable	Delivering gifts to pupils	Importance of relationships	Returning to school after Easter	Finding a way
3	Female Primary SLT	Family member ill with COVID‐19	Finding a way	Working effectively on day of lockdown	Finding a way	Switching to packed lunches	Finding a way
4	Female Primary SLT	Difficult conversation	Teacher identity	Identifying roles for each member of staff team	Importance of relationships	Rude email	Importance of relationships
5	Male Primary SLT	Being sent home from school because clinically vulnerable	Uncertainty	Creating video resources	Finding a way	None identified	N/A
6	Female Secondary SLT	Telling Year 11	Uncertainty	Delivering food parcels to families	Importance of relationships	Realization that it will be ok	Finding a way
7	Female Secondary SLT	Criticism from a parent	Importance of relationships	Staff briefing	Importance of relationships	Governors meeting	Finding a way
				Delivering food parcels	Importance of relationships		
8	Male Secondary SLT	Pupils not engaging remotely	Uncertainty	Pupil sending great video	Finding a way	Decision to prioritize staff welfare	Finding a way
9	Male Secondary SLT	Year 12 leaving children’s home	Worry for the vulnerable	Pupils engaging with new resources online	Finding a way	Engagement with Year 12 girls who were struggling	Finding a way
10	Female Primary ECT	Cancelling school trip	Uncertainty	Den building in school	Reflections	Realizing not all activities are essential	Reflections
11	Female Primary ECT	Notification that a child (not previously known to be unsafe) was unsafe at home	Worry for the vulnerable	Unexpected child engaging well from home	Reflections	Input and engagement from pupils and parents	Finding a way
12	Female Primary ECT	Vulnerable child not attending school	Worry for the vulnerable	Families engaging via new Facebook page	Importance of relationships	Delivering food parcels	Importance of relationships
13	Female Primary MCT	Announcement of lockdown	Uncertainty	First day back in school	Importance of relationships	Establishing a rhythm	Finding a way
14	Female Primary MCT	Announcement of lockdown	Uncertainty	Families engaging via new Facebook page	Finding a way	Adjusting to working from home	Finding a way
15	Female Primary LCT	Announcement of lockdown	Uncertainty	Seeing vulnerable child online	Importance of relationships	Bad day – all too much	Finding a way
16	Female Secondary ECT	Announcement of lockdown	Uncertainty	Creating engaging resources	Finding a way	Being given a clearly defined task to do	Finding a way
17	Female Secondary ECT	Criticism from a parent	Importance of relationships	Appreciation from a parent	Importance of relationships	Progressed to RQT	Teacher identity
18	Male Secondary ECT	Telling Year 11	Uncertainty	Engagement from unexpected students	Importance of relationships	Predicting GCSE grades	Importance of relationships
19	Female Secondary MCT	Saying goodbye to Year 11	Uncertainty	Experience of flexibility	Reflections	Almost empty buses arriving after lockdown	Finding a way
20	Female Secondary MCT	Between school ski trip and lockdown	Uncertainty	Teaching community sharing resources etc	Importance of relationships	Lack of student engagement	Uncertainty
21	Male Secondary MCT	Online choir rehearsal	Uncertainty	Engagement from unexpected student	Importance of relationships	Input from wider teacher community	Importance of relationships
		Rude message from pupil	Importance of relationships				
22	Female Secondary MCT	Not being able to say goodbye to Year 11	Importance of relationships	Nice letters from students	Importance of relationships	Student engagement	Importance of relationships
23	Female Secondary LCT	Sent into isolation due to medical condition	Uncertainty	Online socializing with colleagues	Importance of relationships	Return to school after Easter	Finding a way
24	Male Secondary LCT	Overwhelming instructions from SLT	Uncertainty	Getting the hang of remote teaching	Finding a way	Realizing lessons need to be less complex	Finding a way

Note

ECT = Early Career Teacher; LCT = Late Career Teacher; MCT = Mid‐Career Teacher; SLT = Senior Leadership Team;

We have 25 low point and high point scenes as, in both cases, one participant described two separate scenes. We had 23 turning point scenes because one participant was unable to identify a turning point in their experience to date.

Our sample of primary school teachers consisted of five members of Senior Leadership Teams (SLTs)—who are primarily responsible for strategic planning and the day‐to‐day management of the school—and six classroom teachers, with 1–32 years of teaching experience (*M* = 12.55, *SD* = 8.94). Participants taught in schools in Cambridgeshire, Cheshire, Derbyshire, Hertfordshire, North and East Yorkshire, and Nottinghamshire. Of our 11 primary school participants, 10 were female and the only male teacher was in the SLT group.

Our sample of secondary school teachers consisted of four members of SLT and nine classroom teachers, with 1–32 years of experience (*M* = 15.15, *SD* = 9.18). Participants taught in Devon, Lincolnshire, North and East Yorkshire, Oxfordshire, and Staffordshire. Of these, five were male (two SLT) and the classroom teachers taught English, History, Psychology, Mathematics, Music, and Religious Education.

A wide range of schools were represented by the teachers in the sample (both primary and secondary), with Index of Multiple Deprivation deciles ranging from 1 to 10.

Participants were recruited via emails to existing networks of personal and professional contacts with no conflicts of interest, and through social media advertising. They were compensated with a £20 retail voucher. The project was approved by the ethics committee of the researchers’ university department.

### Measures

We designed a semi‐structured interview based on Section B of the Life Story Interview (LSI; McAdams, 2008). We adapted the LSI to ask participants to tell us about three key scenes in their experience of COVID‐19 as teachers in England: a low point, a high point, and a turning point. We asked them to describe these scenes in as much detail as possible and to reflect on what their choice of scene might say about them as a teacher, that is, to draw meaning from their own narratives. The interview schedule for this section is available in Supporting Information.

### Procedure

Participants received an email invitation to a virtual recorded interview which took place over Zoom (2020), primarily due to social distancing requirements. Interviews took place between Thursday 23 April and Friday 1 May 2020; that is, 5–6 weeks after schools in England were closed to most pupils. The interview consisted of three sections, and the full interview lasted 48 min on average (*SD* = 16 min). The first and longest section of the interview focused on the stories presented in the current manuscript.

### Analysis

Transcripts of the stories teachers told were analysed using reflexive thematic analysis (Braun & Clarke, 2013). We took an inductive approach because COVID‐19, partial school closures, and lockdown represent an entirely new situation. Although we had some research‐informed expectations about how teachers might be affected, rooted in narrative identity theory and research about stress, coping, and identity, we deemed it appropriate to approach this new situation completely inductively. For similar reasons, our analysis is semantic rather than latent, and essentialist rather than constructionist. That is, we aimed to describe teachers’ expressed experiences, their self‐stories, rather than to identify hidden meanings in what they said.

Our coding process began with both interviewers immersing themselves in the transcripts and making initial observations on potential codes and themes. Once this immersion process was complete, one member of the team coded the full data set. All codes were then collated, along with data extracts to support them, and discussed in detail. We organized our codes into initial themes and, as a team, reviewed the proposed themes and refined them to ensure they represented the data and addressed the research question. The themes were then defined and named before writing up the results.

## Results

Our reflexive thematic analysis identified six themes: (1) *uncertainty*, (2) *finding a way*, (3) *worry for the vulnerable*, (4) *importance of relationships*, (5) *teacher identity,* and (6) *reflections*. These themes combine to tell the story of the 24 teachers’ experiences during the first 5–6 weeks of partial school closures in England. Most low point scenes related to *uncertainty*, while most high point scenes reflected the *importance of relationships* and most turning point scenes were about *finding a way*. A summary of the stories teachers told in relation to each of the scenes we requested (i.e. low point, high point, and turning point) and the themes that best describe them at a holistic level can be found in Table 1.

### Theme 1: Uncertainty

This theme captures the first days after the government’s announcement that schools should close to most pupils, along with ongoing uncertainty about what was yet to come. It is characterized by a powerful shared sense of not knowing what is going on. As Participant 24 put it: ‘I guess it felt a bit like, you know, you’re shown the diagram of how the parachute works and then you’re pushed out of the plane’. In most cases, initial reactions involved negative emotions and a sense of rush and panic, ‘like a rug had been pulled from under you’ (Participant 19). This analogy of a rug being pulled from underneath teachers was also mentioned by two other participants (16 and 23). Participant 13 elaborated on multiple factors contributing to teachers’ overwhelming experience of uncertainty at the start of this period:

So within a 24 hour period, we were trying to cope with the fact that we were going into lockdown; cope with the fact that the schools were shutting but not really know what that meant; not knowing what hours that meant we were working and trying to get our heads around what work we could deliver, very quickly, and still teach the children that were in school that day because it was still open and it was still normal.

However, there was a subset of teachers, mainly those in senior leadership positions, who welcomed the initial uncertainty as a challenge. Participant 2, for example, said: ‘…it was all very strange but quite exciting because it was new’. Teachers in this situation described a sense of accomplishment in rising to the challenge that they were faced with: ‘Myself and the Head sat together most of the day and we absolutely nailed what we needed to do’ (Participant 3).

The theme of *uncertainty* has its roots in the fact that schools were told they would be closing to most pupils at the same time as everybody else: ‘So it wasn't like the school could prepare them for anything that happened’ (Participant 22). Teachers were confronted by distress in their pupils and were bothered by not being able to answer their questions.

I'm so used to being almost like a crutch for the children to lean upon. We are meant to know what we're doing. We are meant to reassure them. We are meant to be the kind of guiding light when they're going through … those teenage years which are troublesome sometimes and, and I wasn't able to do that. (Participant 16)

The speed at which schools and teachers were required to enact major operational and emotional changes led to a strong emphasis on *uncertainty*, represented by the prevalence of low point scenes that align with this theme.

The theme of *uncertainty* also reflects concerns about an ongoing lack of clarity from the government that makes it hard to plan ahead. A lack of clear government guidance was felt most keenly by those in senior leadership roles who expressed concern about whether they would be given enough notice of schools re‐opening. There was a sense that the government needed to consult more widely. As Participant 4 put it: ‘I sometimes wish that … the DfE … would listen to all the voices … or a representative sample of voices. Not just the people who shout loudest’. Teachers – senior leaders in particular – want to be consulted on decision‐making that relates to schools, and they want decisions and guidance to be made available to them first so they can prepare to answer the questions their communities will inevitably ask of them.

### Theme 2: Finding a way

*Finding a way* incorporates participants’ accounts of how, once the initial uncertainty had subsided, they adjusted their thinking and behaviour to provide remote education that would meet the needs of their communities, while preserving their own well‐being and that of their pupils.

One common catalyst for teachers relaxing into the situation was seeing evidence that pupils were engaging with the learning they had set for them and that their families were supporting them. ‘And then when I saw it happening, I saw the work coming on, it just made me relax’ (Participant 11). In a handful of cases, pupils appeared more engaged than they would be at school, indicating that the home learning environment might be an advantage for some. Participant 11 shared an example of a 5‐year‐old girl who:

looked so happy to be there and doing work and challenging herself. And I just thought, this isn’t a situation that I’d want for children but for some children, being at home and learning in that environment that they love is clearly working … wow, this could turn a child that doesn’t like school into somebody that really enjoys their education.

Other teachers had a harder time in the initial phases of remote teaching because pupils did not engage with the activities they had created, leading them to rethink their approach. Solving these problems led to a sense of competence, relief, and reflectiveness: ‘what we did to begin with isn't working, so we adapt and change, and I think that's a positive model moving [forward]’ (Participant 20).

Some teachers complained that, during the initial period, their SLTs had inundated them with paperwork and continuing professional development (CPD) activities and that they only began to adjust and find a rhythm that worked once that pressure subsided.

SLT have probably seen that it's not quite as easy as they first thought. And they've changed, I guess, their way of doing things and that's kind of changed things for me as well’ (Participant 14).

Some SLTs, however, recognized this issue early on and prioritized staff well‐being with a strategic view to avoiding burnout. Participant 6 said:

So, there’s all this panic and noise. And then things start to settle, and you start to say, right, okay … we need to be sensible and make sure we protect both staff and students… so that we don't have burnout with both people at home and with staff.

Teachers described a need to create a new work pattern that would allow them to do their job and protect their well‐being, especially as the boundaries between work and home became blurred. They described the need to incorporate competing responsibilities, such as teaching their own children, into that new routine.

we've got our own children at home, who, you know, you want to help, and they're getting all their work set for them. And, you know, obviously, the teacher in me is going ‘they need to do the work …’ and the Mum in me is going, ‘they're getting completely stressed out. We need to go for a walk and jump in puddles for a bit’. (Participant 15)

Several participants acknowledged that the situation was likely to be hardest on those with young children. That said, some teachers who lived alone reported feeling lonely or isolated, and needing to proactively put measures in place so that they did not work all of the time.

### Theme 3: Worry for the vulnerable

Almost all participants expressed concern for vulnerable pupils, particularly those known to be unsafe in their own homes. This issue appeared to generate more anxiety and sadness in teachers than any other. They were concerned about these pupils from the outset of school closures but, once they had found their feet in navigating remote education, concern for vulnerable pupils became their main priority. There was strong evidence of a desire to get ‘eyes on the child’ after, for example, being notified that a child had witnessed domestic violence (Participant 2). Participant 15 said teachers are:

used to seeing them so regularly and … being able to check in … and know, yeah, they're okay; they've had … something to eat, they've got… clean clothes … all those little things that you just do … to just check in on a child, and you do it a million times a day when you're in school. But not having that contact at all …

Teachers’ narratives suggest that Designated Safeguarding Leads (DSLs), who are on the frontline of school support for ensuring the pastoral well‐being of the most vulnerable pupils, may need more support than they are currently receiving, and better coordination between national government, local government, and schools. They may benefit from a system that allows them to share some of the distressing encounters their job entails with a colleague or another DSL so they can be emotionally and practically supported at a time when their role is harder than ever.

Participants, particularly members of SLTs, referred to pupils living in families who did not have enough to eat. Participant 4 described how ‘the free school meals portal with Edenred is a disaster’, referring to a situation in which a private company was tasked by the government with providing food vouchers to all families eligible for free school meals but failed to deliver them on time. However, it was notable that in some cases high point scenes involved addressing that problem directly by making up food hampers and taking them to the families that needed them.

When you deliver a bag of food to a family who… literally have nothing … they have lost their jobs because they were zero hours contract workers; they've got three or four children; and we know on a daily basis when they're in school those children are hungry all the time because we see them … it … remind[s] you about society. (Participant 7)

As well as issues of safety and food poverty, teachers pointed out that many families have limited access to the technology required to engage in online learning. Participant 4 said:

if somebody in authority asked them the question, ‘do you have internet access?’ they, of course, they would say yes. But in reality what they have is a phone, that's Mum's phone that she can get the internet on … And Mum is terrified of wasting the data because she's got no money to buy some more.

It was clear in the narratives that this theme of *worry for the vulnerable* works on different levels. Classroom teachers worry about children they have concerns about and, to some extent, their worries are allayed by seeing the child on screen or receiving a piece of work from them. Members of SLTs, especially DSLs, have a particularly difficult job to do as keeping pupils safe requires coordination with social services and clear guidance from the government. In a similar vein, when the government promised food and laptops for those who needed them but found themselves unable to deliver, this responsibility fell on individual schools’ SLTs, creating additional pressure. This appears to have led to anger and frustration on the part of schools and teachers but our data suggest that these emotions are overshadowed by the worry that they feel about the children who would normally be in their care five days per week.

There was a strong sense that the pandemic is highlighting broader problems that were always there, just hidden. As Participant 1 said:

I think it was the fact that the government then decided to give free school meals during the holidays. And that to me was, ‘but we don't normally’. So your first thought is ‘why are we doing it in the holidays?’ But … when parents weren't getting it, and you know … these are proud parents who are then ringing me up and saying, ‘I don't have the voucher’ … this is an issue ongoing. And I think, you know, one of the things I want to be doing is kind of petitioning people saying, you've got to look at what we did in this event… these people need this support year in year round, not just because we're in a pandemic.

In summary, this theme of *worrying for the vulnerable* captures a very high level of anxiety about disadvantaged pupils that was common to teachers at all levels of experience. Teachers described feeling powerless to help pupils they are used to looking out for and worried about those whom they may not even have realized were at risk before now. It also captures the idea that the vulnerabilities that are coming to light have always been there and that the current lockdown situation is merely highlighting bigger social issues.

### Theme 4: Importance of relationships

Teachers’ narratives illuminated the fundamentally social nature of teaching. As Participant 23 put it: ‘we’re all fairly social creatures’. Participant 21 said: ‘everything that you do in the classroom is about building trust in each other’. They described sadness at the abrupt ending of relationships with pupils in Years 6, 11, and 13. Participant 16 said: ‘it just felt like I’d been ripped apart from my career almost because I couldn’t see the kids. I couldn’t say goodbye to them’. Participants also reported distress and concern about disrupted relationships, for instance where pupils were not engaging or where pupils and their parents provided negative feedback. As Participant 17 put it, in describing a complaint from a parent:

I don't really have that thick teachery skin yet. So little things like that can really get to me… I just felt really bad about it … you can't always prepare for every single scenario in two days before you're kicked out the building.

However, in spite of moments of sadness and concern about relationships, this theme was mainly characterized by the protective nature of relationships. Participants talked about how highly they valued their relationships with pupils and their families. Participant 6 said: ‘staff and students invest in each other really’, and Participant 12 described setting up a Facebook group that successfully engaged families: ‘I think it’s just making sure I know for myself I’ve done my best to communicate with parents’. Above all, it was clear that teachers valued their professional relationships with their colleagues and that, in many cases, these professional relationships had developed into genuine friendships which have been an enormous source of support. Some participants reported online professional and social events as high points and classroom teachers mainly felt that the support they need rests within their school, and primarily in their colleagues. Participant 7, in describing a staff meeting, said: ‘One of them was a member of staff who was in a fancy dress and it made people smile and it was a simple, tiny moment of, of joy in quite a complex time’. Some participants also described a strong wider teaching community held together by social media. ‘So I think a huge high point is how the teachers in the profession have really rallied around each other’ (Participant 20).

It is notable that many participants’ low point scenes involved disrupted relationships while many high points were stories of strong and supportive relationships with pupils, families, and other teachers.

### Theme 5: Teacher identity

In telling their stories, participants talked about how their professional identity had been affected. They often referred to what they saw as widely shared teacher characteristics, such as caring for pupils and wanting to be in the classroom. Teachers’ need for organization and desire to plan were also apparent; as Participant 5 put it: ‘I think if you ask any teacher they like their routines and structures’. As there have been many uncertainties associated with the COVID‐19 situation, ‘this has really, really thrown us … by nature we're sort of planners’. (Participant 2).

A strong sense of a shared teacher identity was used to explain reactions to the scenes that participants narrated. Several described their dismay at their job becoming an ‘admin job’ and how remote interaction or being in school helped to reduce this feeling. Participant 22 said: ‘I think, you know, that's that's good to be able to do in a way. You feel more like a teacher than somebody that's just providing resources for students’. Members of SLTs tended to go into school more often than classroom teachers, and all expressed pleasure in having that opportunity. Teachers also talked about their desire, or need, to ensure pupils were treated fairly and this was felt particularly strongly by those required to grade national assessments. Participant 9 said: ‘I don’t think anyone will ever appreciate the amount of effort that’s gone into getting these grades right’. Participant 18 was concerned that GCSE pupils from low performing schools would be disadvantaged by the system: ‘our school is … it’s in the bottom 10% of all schools, you know, in terms of Progress 8 so I think students that would get a 9 in their exam … I don’t think they will …’

In summary, teachers drew upon characteristics they perceived as being widespread in the teaching profession to find ways to make remote education work for them and their pupils. That is, expressions of the core characteristics of the profession were prevalent, including a need for routine and planning, caring about pupils and wanting to be in a classroom, interacting with others, and doing their job.

### Theme 6: Reflections

Although lockdown presents major challenges to the teaching profession, after the initial shock many participants began to reflect on their new circumstances and to find some silver linings. Most said they are now less busy and pressured than before and that they have the opportunity to use the extra time available to them in beneficial ways: ‘we've actually been able to use the time to plan appropriately for next year, which we often can't’. (Participant 20).

There were important caveats to this though, namely that work–life balance has become more blurred, with some teachers unable to take breaks such as the Easter holiday. In spite of this, most participants said they appreciated having more manageable hours and the opportunities this gave them for curriculum planning and reading. As Participant 19 put it: ‘I’ve been able to get up really early, get loads of stuff done. And then it’s still daylight, it’s nice weather … you can go out and exercise in the afternoon’.

Having flexibility and freedom from the national curriculum was also seen by many as an upside, allowing teachers to be creative and to differentiate in meaningful ways. Participant 10 describes a day in school with a small group of key workers’ and vulnerable children when ‘we went into den building and we did some like whistling as well, which was quite good. And yeah, we've been getting away with doing a bit more risky stuff I guess’. Participant 13 said: ‘I had more time to spend chatting to the children and helping them on more of a one to one basis than you would ever have’. Another upside noted by participants was the emergence of pupils who may be considered ‘surprise stars’. These were pupils who had not shone in the classroom but stood out while working from home. For example, Participant 18 described his experience with a pupil who had proactively asked for work, submitted it and then asked for more, commenting:

And I was kind of like, wow, this is like, you know, he’s quite a weak student academically but yet he was willing to try… And in a strange way you can actually build a relationship with some of the more difficult students because their life is so difficult … they’re almost looking for some kind of escape mechanism.

Another participant, with a similar story commented: ‘I think I’ll approach that student in a very different way now’ (Participant 21). Against the backdrop of challenges introduced by partial school closures, most participants were able to identify professional or personal benefits once the initial panic had subsided.

Participants described using this period to reflect on whether some of the changes might be incorporated into post‐COVID‐19 education. Participant 8 reflected on how teaching may become more technologically oriented and how ‘…in school…the old method of ‘stand at the front, dictate to the class’, I think, is on its way out’. For some participants, these reflections were tied up with their worry for the vulnerable and a renewed understanding of the barriers pupils face. Participant 1 said: ‘it’s been about different priorities … we’re kind of in the Ofsted window and you get a bit bogged down and actually, for me, it’s helped to prioritise what’s really important’. This led several participants to consideration of more holistic approaches and their implications for learning, assessment, and well‐being.

## Discussion

We identified six themes in teachers’ stories of low points, high points, and turning points during the first 5–6 weeks of partial school closures and lockdown in England: *uncertainty, finding a way, worry for the vulnerable, importance of relationships, teacher identity,* and *reflections*. By asking participants to share scenes from their experiences, we were able to gain insight into their narrative ‘teacher‐selves’, and the meaning they drew from the experiences they described. Here, we discuss our findings and draw out implications for teachers, school leaders, and policymakers.

### Teacher stress and coping

The major teacher stressors are usually workload and behaviour management (Catalán *et al.*, 2019; Boyle *et al*., 1995; Kokkinos, 2007). By contrast, the participants in the current study focused on two rather different stressors. The first was uncertainty related to the announcement of partial school closures, navigating immediate demands, and planning for what might happen next, as UNESCO (2020b) forecasted. The second stressor concerned worry for vulnerable pupils and, in many instances, their families. Although workload was mentioned by some, it was not a prominent feature of the teachers’ stories and behaviour management barely featured at all, perhaps for obvious reasons.

In line with the conceptualization of stress by Lazarus and Folkman (1984), appraisal and interpretation of the same stressors indeed seemed to differ between individuals. First, while some teachers initially appraised the changes around them as overwhelming, others appraised them as an exciting, novel challenge to overcome. Moreover, teachers said their initial stress was substantially alleviated by finding a way forward but some, particularly those in senior leadership positions, remained stressed about a lack of clarity around the government’s short‐ to medium‐term plans. In this light, government consultation, clarity, and realistic timeframes for change will be essential to relieving teachers’ stress in the coming months.

Schools and teachers have long acted as an important safety net for pupils (Lasky, 2005), and the current situation has highlighted the vulnerable conditions in which some children and young people live. The difficulties teachers experienced in reaching ‘at risk’ or disadvantaged pupils led to them feeling powerless to help those who may need them the most. With reports of a rise in domestic violence and growing job losses, alongside problems with ensuring families have sufficient food and access to technology, teachers’ worries appear to be warranted (OECD, 2020c). Teachers’ usual safeguarding mechanisms have been taken away, and, without ‘eyes on the child’, many find themselves in the stressful situation of being unable to protect their pupils. Solving this problem is likely to involve a more nuanced approach and better links between education, social care, and health services than was evident in our data. Addressing this represents an urgent challenge for vulnerable pupils but also for their teachers and schools.

When teachers found the uncertain situation in which they found themselves stressful and overwhelming they seemed, initially, to use problem‐focused coping strategies to manage the immediate and most pressing stressor of delivering education remotely. Strategies of active coping, planning, and time management (Carver *et al*., 1989) were particularly apparent. For example, UK teachers have reported their schools quickly finding ways to deliver teaching using a variety of approaches to remote education, ranging from providing work packs (distributed via email or on paper) through to full days of synchronous online teaching (Department for Education, 2020b). It should be noted that none of our participants reported delivering full synchronous teaching; approaches ranged from distributing work packs through to online options including narrated PowerPoints, videos, and access to educational websites. Several participants reported serious practical challenges with online teaching, such as how to communicate with pupils who do not have access to a computer or a quiet learning space (OECD, 2020a). That said, most reported a good level of success with their chosen approaches but noted more engagement from those in higher ability groups, fuelling concern about a growing disadvantage gap. It is important to note that most approaches were put in place as short‐term solutions but that, as it becomes clearer whether some remote teaching will be necessary in the medium and longer term, further development of these approaches may be required. Thus, this may represent a further stressor for teachers to cope with in the coming months, especially if they are also engaged in face‐to‐face teaching.

While dealing with the stress of navigating practical problems, teachers also reported feeling emotionally overwhelmed by the changes they were experiencing. In response, they primarily used emotion‐focused strategies, particularly seeking emotional support from colleagues and venting to each other (Carver *et al*., 1989). In fact, they seemed to express an increased desire for these types of strategies and made extra efforts to create and develop relationships with each other, through both formal and informal means. Interaction with trusted colleagues provided a safe space to offload and support one another as they navigated shared stressors (Hargreaves, 2001), creating a sense of community among teachers that can be considered part of teacher identity (Hanna *et al*., 2019). This sense of belonging and support between teachers is possible if they are familiar with their colleagues. As new teachers are hired within schools and remote teaching may become necessary again, extra support for these teachers may be beneficial such that they too can draw on colleague support in the future. All in all, the camaraderie between teachers within the school and within the profession may be an important resource that teachers can continue to use as they navigate the affective changes associated with the changes brought about by COVID‐19.

### Core values in teaching

Working from home and delivering remote education were described by some as being harmful to their professional identity and alien to their core values, making them ‘not feel like a teacher’. Participants’ stories highlighted shared values, such as being caring, fair and social, and needing structure, routine, and the capacity to plan.

Teachers have a basic need for relatedness (Catalán *et al.*, 2019), and this was evident in stories which featured their relationships with pupils, parents, and colleagues. Establishing positive teacher–student relationships is not only fundamental to their own well‐being (Spilt *et al*., 2011) but also their pupils’ self‐esteem, well‐being, and engagement (Lavy & Naama‐Ghanayim, 2020). Teachers are known to find meaning and purpose in these relationships (Veldman *et al*., 2013), and an extended remote teaching experience could have implications for job satisfaction and attrition, which is a real concern given the teacher shortage in England (Department for Education, 2019b). Moreover, we found evidence of an increased level of trust between parents and teachers. Teachers reported some parents doing a good job of supporting their children, and this allowed many of them to relax and focus on the way forward. Enhanced parent–teacher relationships and a sense that parents and teachers are on the same team may prove beneficial when pupils return to school. Additionally, relationships with colleagues are an important element of being a teacher. As discussed above, seeking emotional support from colleagues was a common way for participants to cope with stress. Thus, it seems that the core values of the profession, and the way teachers cope with stress, can go hand in hand in some respects, which further emphasizes the importance of teachers establishing strong relationships with their colleagues.

Other shared characteristics were apparent in addition to valuing the social element of their job. Teachers’ stories reflected high levels of caring for pupils and for each other, and a need for their pupils to be treated fairly, particularly in relation to national assessments and having equal opportunities to learn. It seems likely that the uncertainty inherent in the COVID‐19 situation provoked a high level of stress, because it threatens core facets of teacher identity, including a need for routine, structure, and the capacity to plan. Thus, these qualities may be important to consider when defining and understanding the components of what it means to be a teacher (Hanna *et al*., 2019). As this project is part of a longitudinal study, we will be able to track how the narratives participants present change and become internalized into their evolving COVID‐19 stories over the course of the pandemic.

### Implications for policy and practice

Our data suggest that the educational communities represented in our sample adjust well; that teachers support each other effectively and that they support their pupils and benefit, in turn, from the engagement they receive from pupils and their families. Most of the additional support that schools and teachers require is from the government and agencies such as social services and child development professionals. Both UNESCO (2020c) and the International Task Force on Teachers for Education 2030 (2020) have emphasized that teachers should be included in national education‐related decision‐making, and teachers in this study reported that far too little of this had been happening in the current situation. In order to reduce confusion and stress for teachers and to ensure effective and systematic implementation of policy, much more government consultation and communication with school leaders are recommended. Additionally, identifying practical ways of supporting DSLs in providing a strong enough safety net for at‐risk pupils may be needed.

### Limitations and future directions

The current study is cross‐sectional in design and represents only a snapshot of participants’ experiences during the 5–6 weeks after partial school closures. This study forms the first part of the project, which will track these teachers’ stories at three further time points with a view to identifying and understanding their experiences as the situation evolves (Saldaña, 2003). We hope these longitudinal data will highlight essential components of teaching and the profession and how teachers can be supported in the current circumstance and beyond.

Furthermore, one must note the source of our data. We only gathered data on teachers’ experiences so future studies may benefit from triangulating teachers’ experiences with those of their pupils. Also, we focused on teachers in mainstream primary and secondary schools because experiences in independent and alternative provision schools are likely to be different. Future studies could focus on these groups.

### Conclusion

Teachers are the social fabric that holds the educational system together, and it is therefore important to protect their ability and capacity to fulfil their role. To authors’ knowledge, the current study is the first to document teachers’ narrative experiences during the initial period of lockdown in England. Findings highlight the challenges that teachers have faced and lessons that have been learned at this stage of the pandemic. The future, which remains somewhat unclear at the time of writing, will inevitably require great flexibility, resilience, and collaboration from teachers, and the uncertainty involved is likely to continue to be stressful for them. Clear communication from the government regarding future plans and joined‐up thinking around supporting the most vulnerable pupils will be key to protecting teachers. By listening to teachers’ self‐stories at this seminal point in time, we can gain new insights into what it means to be a teacher and how teachers can be supported to work in ways that make their job fulfilling and benefit their pupils and the communities that they serve.

## Conflicts of interest

All authors declare no conflict of interest.

## Author contributions

Lisa E. Kim, Ph.D. (Conceptualization; Data curation; Funding acquisition; Investigation; Methodology; Project administration; Writing – original draft; Writing – review & editing) Kathryn Asbury (Formal analysis; Funding acquisition; Investigation; Methodology; Writing – original draft; Writing – review & editing).

## Supporting information


**Appendix S1.** Life stories section of time 1 interview schedule.

## Data Availability

Data can be made available to researchers by contacting the corresponding author.
