# Supplementary material for: ‘Like a rug had been pulled from under you’: The impact of COVID‐19 on teachers in England during the first six weeks of the UK lockdown
Source: Br J Educ Psychol. 2020 Sep 25;90(4):1062–83. doi: 10.1111/bjep.12381 (PMC7537096; doi:10.1111/bjep.12381)
Supplement: Supplementary file 1 — Appendix S1. Life stories section of time 1 interview schedule. [file BJEP-90-1062-s001.docx]

**Supplementary Material**

**Life Stories Section of Time 1 Interview Schedule**

I would like you to start by telling me the story of your experience so far of being a teacher/headteacher during the coronavirus pandemic. I am going to ask you to describe some scenes from your story, a low point, a high point, and a turning point.

1a. First of all, please can you tell me about the **low point** in your experience of being a teacher/headteacher during the pandemic so far? Even though this event is likely to be something unpleasant I would appreciate you telling the story of this low point in as much detail as you can. For example, what happened, when and where, who was involved and what were you thinking and feeling?

1b. Why do you think this particular moment was so bad, and what does it say about you as a teacher/headteacher?

2a. Now I would like to ask you to describe a scene that you would describe as a **high point** during your experience of being a teacher/headteacher during the pandemic so far. Please try to describe this high point scene in detail. Think about what happened, when and where, who was involved, and what were you thinking and feeling? Please tell the story of your high point in as much detail as you can.

2b. Why do you think this particular moment was so positive, and what does it say about you as a teacher/headteacher?

3a. The final scene I would like you to describe is a **‘turning point’** scene. In looking back over your experience of the pandemic so far can you identify a scene that stands out as a ‘turning point’ for you as a teacher/headteacher? If you cannot identify a key turning point please describe an event from the past month where you went through an important change of some kind. Again, for this event please describe what happened, when and where, who was involved and what you were thinking and feeling.

3b. As before, please can you say a word or two about what this ‘turning point’ event says about you as a teacher/headteacher, or about your teaching career.
